# The dynamics of meaningful social interactions and the emergence of collective knowledge

**DOI:** 10.1038/srep12197

**Published:** 2015-07-15

**Authors:** Marija Mitrović Dankulov, Roderick Melnik, Bosiljka Tadić

**Affiliations:** 1 Department for Theoretical Physics, Jožef Stefan Institute, Ljubljana, Slovenia; 2 Scientific Computing Laboratory, Institute of Physics Belgrade, University of Belgrade, Belgrade, Serbia; 3 MS2Discovery Interdisciplinary Research Institute, M2NeT Laboratory and Department of Mathematics, Wilfrid Laurier University, Waterloo, ON, Canada

## Abstract

Collective knowledge as a social value may arise in cooperation among actors whose individual expertise is limited. The process of knowledge creation requires meaningful, logically coordinated interactions, which represents a challenging problem to physics and social dynamics modeling. By combining two-scale dynamics model with empirical data analysis from a well-known Questions & Answers system *Mathematics*, we show that this process occurs as a collective phenomenon in an enlarged network (of actors and their artifacts) where the cognitive recognition interactions are properly encoded. The emergent behavior is quantified by the information divergence and innovation advancing of knowledge over time and the signatures of self-organization and knowledge sharing communities. These measures elucidate the impact of each cognitive element and the individual actor’s expertise in the collective dynamics. The results are relevant to stochastic processes involving smart components and to collaborative social endeavors, for instance, crowdsourcing scientific knowledge production with online games.

In modern statistical mechanics^1^, it has been recognized that the collective phenomena arise from interactions among the elementary units via a spontaneous transition to an organized state, which can be identified at a larger scale^2^,^3^. Recently, this unifying principle is gaining importance in other natural sciences, for instance for elucidating organization in living systems^4^,^5^,^6^,^7^,^8^, emergence of coherent activity in neuronal cultures^9^, and developing computational social science^10^. In social systems, interactions and cooperations among actors can lead to the recognizable collective behavior, for instance, the development of collective knowledge^11^, appearance of common norms^12^ or language^13^. The quantitative study of the stochastic processes underlying these social phenomena utilizes the methods of statistical physics supported by analysis of the plethora of online empirical data. Some illustrative examples are the appearance of good and bad conduct in online games^14^ and groupings induced by the exchange of emotional messages on social sites^15^,^16^,^17^,^18^. However, a deeper understanding of the mechanisms of collaborative social endeavors^11^,^19^,^20^ remains a serious challenging problem in physics and social dynamics modeling.

The building of collective knowledge via social interactions is a subtle phenomenon that requires both cognitive elements and an organized effort to solve a particular query. In this stochastic process, the social system that enables transfer of knowledge and the cognitive subsystem are dynamically interlinked and influence each other at a microscopic scale^21^. In the relational epistemology, the exchange of values is an essential factor that permits the emergence of a collective value via interaction and cooperation among equal individuals^22^. In this concept, the collective knowledge is neither an entity over individuals nor their sum, rather, it is a property of the particular relations among the interacting actors. It reflects the actions of each individual as a *meaningful, adjusted to the actions of others by means of new operation*; its reciprocity and the acceptance of the confirmed values lead to a cooperation “that has a logical structure isomorphic to logical thought”^22^. On the practical side, modern information communication technologies (ICT) provide a suitable platform for knowledge building via social dynamics^23^,^24^. These systems aim at transferring the expertise and tacit knowledge that reside in the minds of individuals into a form of collective knowledge. Through ICT, the individual’s knowledge is shared or “externalized”^21^. Also, the fragile relational state, where the knowledge is dynamically experienced within a community, is actualized as a collection of mutually related digital artifacts. When a systematic tagging is applied to these artifacts, a form of “explicit” knowledge appears, from which others can learn^21^. For this reason, the emergence and quality of the collective knowledge crucially depend on the microscopic mechanisms, by which a particular cognitive element and an individual actor’s expertise contribute to the self-organized process.

We develop a new approach that explains how the collective knowledge emerges in Questions & Answers (Q&A) communications. We utilize the concept of two-scale dynamics that enables defining a correct microscopic model of interactions between social and cognitive elements, and confirm its predictions by quantitative analysis of the empirical data from a well-known Q&A system. The elementary units in the process, actors, and questions that they post or answer contain sub-elementary units—cognitive elements, which describe the actor’s expertise and the questions’ cognitive contents. Their dynamics strictly obeys the cognitive recognition rules, thus influencing the dynamics at the social level of actors. We quantitatively describe the knowledge-creation process from the elementary interactions to mesoscopic and global level. The statistical signatures of the collective dynamics depend on the range of the actors’ expertise, which can be extracted from the empirical data and varied in the simulations. The impact of cognitive elements in the empirical data is further confirmed by methods of information theory while the occurrence and structure of communities are visualized by graph theoretic techniques.

## Results

### Fine-grained dynamics and cooperation

All our exemplifications are provided based on the analysis of data in mathematics from the system known as **Mathematics** which has become a universal clearinghouse for Q&A in the field^25^. In the data, the cognitive element of each artifact (question, answer or comment) has been systematically tagged according to the standard mathematics classification scheme. In addition, the fact that a unique identity is known for each actor (user) and each artifact together with the high temporal resolution of the data enable a detailed analysis of the underlying stochastic process. Assuming that the cognition-driven events occurred, we determine a set of tags as expertise of each user in the considered dataset. The dataset and the procedure are described in Methods. In the model (Supplementary information, SI), the actors (agents) have a defined range of expertise. Minimal matching of the expertise of an answering agent with the tags of the answered question is strictly obeyed. The considered agents have the activity patterns statistically similar with the patterns of users in the empirical data while their expertise is varied.

**Figure 1:**
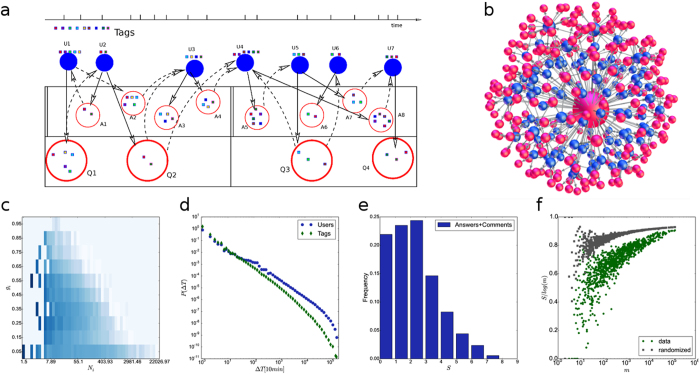
Tags-matching illustration and the activity patterns of users and tags in Mathematics. (**a**) Schematically shown a sequence of events with matching of tags (colored boxes) between actors’ expertise (displayed as a particular set of tags above blue circles—actors, *U*_*i*_), the answers *A*_*j*_, and questions *Q*_*j*_ containing the tags of the related actor’s expertise. The direction of lines towards/outwards each actor indicates the process of reading/posting event. (**b**) Bipartite network of users (blue) and answers (red) at a favorite question (big red node). (**c**) Probability *g*_*i*_ of posting a new question by the user *i* plotted against its total activity *N*_*i*_, averaged over all users in the dataset. (**d**) The distributions of the interactivity time Δ*T* for users and tags. (**e**) The distribution of the user’s expertise entropy *S*_*i*_ averaged over all users in the data. (**f**) Each point indicates the entropy related with the probability of the appearance of a particular tag along a sequence of *m* time intervals, where *m* is the tag’s frequency. Lower set of points represents the entropies for all tags computed from the sequence of events in the empirical data while the upper set is obtained from its randomized version.

In the process, which is schematically depicted in Fig. 1a, an actor (U) posts a question (Q), which may receive answers or comments (A) by other actors over time. Subsequently, new Q and the already present Q&A are subject to further answers, and so on. Representing each action by a directed link, this process co-evolves a bipartite network, where actors are one partition and Q&A form another partition. An example of a single-question network from the empirical data is shown in Fig. 1b. The cognitive content of each question is marked by up to 5 different tags, which thus specify the required expertise of the answering actors. Matching by at least one tag is required. The actor’s expertise is transferred to its answer. The excess expertise of the involved actors leads to the innovation^26^,^27^,^28^ and an accumulation of expertise around a particular question. At the same time, it extends the sample space of matching events, thus accelerating the process in a self-organized manner.

**Figure 2:**
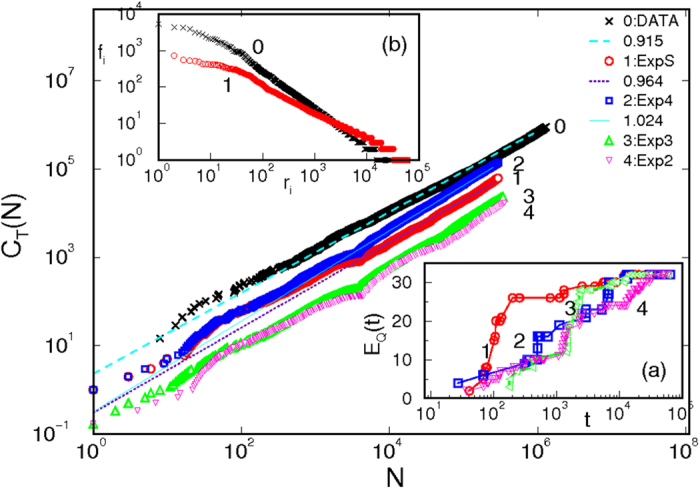
Innovation growth by the actor’s expertise. Main panel: The number of new combinations of tags *C*_*T*_(*N*) at questions including answers to them is plotted against increasing total number of artifacts *N*. The curves 0 · · · 4 are for the empirical data and simulations where the number of the agent’s expertise is fixed as follows: (ExpS), 2^*S*^-tags expertise where *S* is taken from the distribution in Fig. 1e, and (Exp*n*), *n*-tags expertise where *n* = 4, 3, 2. Inset (**a**) Increase of the knowledge at a particular question *E*_*Q*_(*t*) over time *t* for diverse distributions of expertise as in the central panel. Inset (**b**) Ranking distribution for frequency of new combinations of tags appearing in questions and the related answers for (0) the empirical data and (1) simulation in the case ExpS.

The quantitative measures displayed in Fig. 1(c–f) signify a highly cooperative process with the cognitive elements encoded by tags in the empirical dataset. Specifically, the entropy in Fig. 1f shows a distinctly non-random pattern of the appearance of each tag. In accordance with the entropy, the use of different contents shows temporal correlations. The distribution of time intervals between consecutive events with a particular tag ranges over five decades, Fig. 1d, suggesting a variety of roles that different cognitive elements play in the process. The dynamics of tags closely reflects the heterogeneity of the users’ activity profile and their expertise. Figure 1d also shows the broad distribution of the interactivity time of a particular user; the presence of a daily cycle is characteristic of online social dynamics^15^,^17^. The long delays between actions of some users, contrasted with a frequent activity of others, yield the power-law distribution of the number of activities *N*_*i*_ per user (Fig. 3a in SI). Further, the role of each user in the process can be distinguished. For instance, in Fig. 1c, the probability for posting questions *g*_*i*_ decays with the number of the user’s actions *N*_*i*_. Essential for the cognitive process, however, is the broad range of the user’s expertise. As discussed in Methods, it is measured by the entropy distribution shown in Fig. 1e. While the majority expertise includes between one and four tags, few individuals have an activity record for a large number of topics. Consequently, the appearance of a particular combination of cognitive elements shows a complex pattern. All distinct combinations of tags found in the dataset obey Zipf’s law, see Fig. 2. It is a marked feature of scale-invariance in the collective dynamics^28^,^29^. The ranking distribution of individual tags is also broad, Fig. 2 in SI. Furthermore, by directly inspecting the related time series, Figs 4 and 5, we find that an actively self-organized social process underlies the observed dynamics of cognitive elements.

### Advance of innovation

In the present context, the innovation is measured by appearance of new combination of tags *C*_*T*_(*N*) with the addition of artifacts *N*, Fig. 2. The universal Heaps’ law, *C*_*T*_(*N*) ~ *N*^*α*^, and the related^29^ Zipf’s law shown in the inset (b) of Fig. 2, obtained from the empirical data are supported by the simulations. Here, the innovation is directly given by the excess expertise of the active agents. Thus, the accumulation of expertise at a given question depends on the population of experts; it is slower when, e.g, each agent has two-tags expertise than in the case of four-tags expertise, inset (a) of Fig. 2. In contrast, no increase in innovation is observed when each agent possesses a single-tag expertise.

### Information divergence

**Figure 3:**
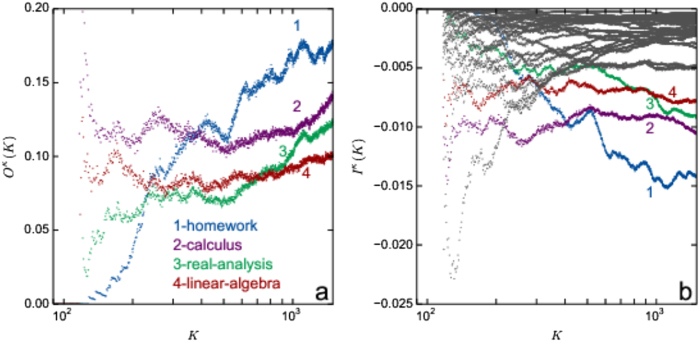
Measuring the impact of a particular cognitive content (***κ***-tag). Likelihood *O*^*κ*^(*K*) for four most active tags (**a**) and Information divergence *I*^*κ*^(*K*) for 30 most active tags (**b**) are plotted against the time-window index *K*.

To examine the influence of a particular cognitive element (tag) in the process, we define a set of conditional probability measures and compute the discrete Kullback-Leibler information divergence from the sequence of question-answer events in which that tag is present, Fig. 3. The empirical data are divided into a series of one-day time windows. In what follows, we use the time window index *K*, which runs in our examples as *K* = 1, 2, · · · 1498. As the activity on a particular question or answer typically extends over many time windows, for *K* ≥2 the space of events *Q*^*K*^ for questions in the *K*th window also includes Q&A which were active in the (*K* −1)th window, while only new answers in the *K*th window make the sample space *A*^*K*^ for answers. By focusing on the time-line of the tag *κ*, which annotates a particular cognitive content, four conditional probabilities, which are defined in Methods, are determined in every time window *K*. The information divergence^30^,^31^,^32^, defined as *I*(*P*(*κ*|*A*^*K*^, *Q*^*K*^)||*P*(*κ*|*Q*^*K*^)) ≡  *I*^*κ*^(*K*) within the time window *K*, is computed by


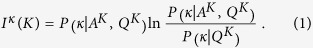


It determines the information gain about the *κ*-tag that is present in questions *Q*^*K*^ if the answers *A*^*K*^ are known. Using the chain relation *P*(*κ*|*A*^*K*^, *Q*^*K*^)*P*(*A*^*K*^|*Q*^*K*^) = *P*(*A*^*K*^|*κ*, *Q*^*K*^)*P*(*κ*|*Q*^*K*^), it can be expressed as *I*^*κ*^(*K*) = *P*(*κ*|*Q*^*K*^)*O*^*κ*^(*K*) ln *O*^*κ*^(*K*) or:


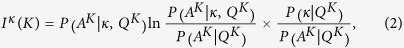


where the ratio 
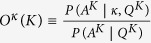
 is a measure of the likelihood that the presence of the *κ*-tag in questions triggers answers within the time window *K*. We compute *O*^*κ*^(*K*) for four most frequent tags, Fig. 3a, in the sequence of time windows *K*. A significant difference among tags is apparent; for instance, “real analysis” triggers more activity than “linear algebra”, but still less than “calculus” and “homework” tags.

In view of Eq. (2), the information divergence is expressed (apart from a multiplicative factor smaller than one) as the negative of a relative entropy, which measures the information loss when the probability of answers to questions containing a given cognitive content *κ* is approximated by the probability of answers to all questions. This probability is expected to increase with the accumulation of expertise around each question over time. Consequently, the information divergence tends to zero for a sufficiently large time. *I*^*κ*^(*K*), computed for 30 leading tags in the empirical data, Fig. 3b, levels to zero for the majority of tags at large *K*. However, in the case of four tags, for which the increase in the likelihood of new activity occurs, Fig. 3a, the information divergence still decreases within the entire time interval in the empirical data, four marked curves in Fig. 3b. Note that these topics of a broad interest often combine with new tags, i.e. via the expertise of new arrivals. In this way, triggered answers that match these new tags expand the sample space *A*^*K*^, which keeps the information divergence finite. This feature is compatible with the innovation growth, reported in Fig. 2. Accounting the contribution of each particular tag in the knowledge creation, the results of information divergence complement the statistical measures in Fig. 1 and support the occurrence of Zipf’s law.

### Signatures of self-organization in the social process

**Figure 4:**
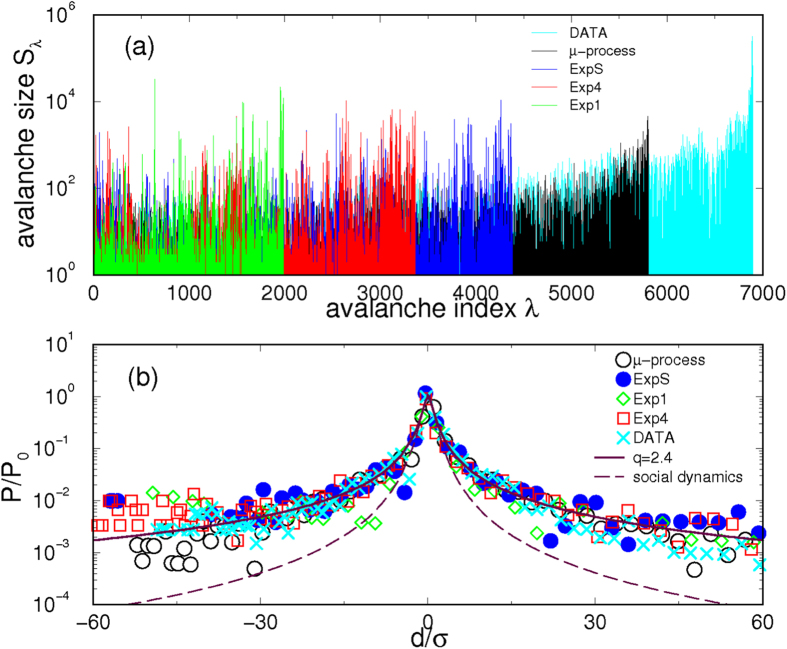
Clustering of events with cognitive contents. (**a**) Sequences of clustered events (avalanches) of the size *S*_*λ*_ against the cluster’s index *λ*. Different colors indicate the sequences identified in the empirical time series and time series simulated for various ranges of the agents’ expertise. (**b**) Distribution of the first returns scaled by the standard deviation *σ* of the corresponding sequence (matching color). Full line indicates *q*-Gaussian curve with the parameter *q* = 2.4. For a comparison, the curve with *q* = 1.8 is shown (dashed line), corresponding to the case of chat channel dynamics studied in^15^.

**Figure 5:**
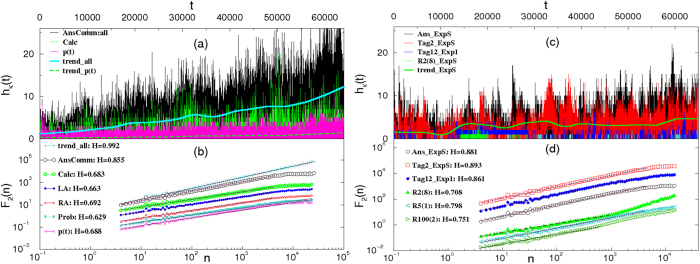
Persistent fluctuations in answering activity in data and in simulations. (**a**) In the empirical dataset, time series of new users *p*(*t*), and time series of the number of answers and comments, and the number of events involving a particular tag (“calculus”). (**b**) The fluctuations *F*_2_(*n*) ~ *n*^*H*^ around the local trend are plotted against the time interval *n* for time series in (**a**) as well as their trends, and time series involving a particular tag: LA—“linear algebra”, RA—“real algebra”, Prob—“Probability”. (**c**) and (**d**) Time series and their fluctuations in the simulations: time series of the number of all answers, and the number of answers containing a particular tag no.2, as well as series containing a particular combination of eight tags R2(8), one-tag, R5(1), and two tags combination, R100(2), all for the distribution of expertise ExpS, and the answers containing tag no.12, in the case of Exp1. Lines are shifted vertically for better display. On each line, the scaling region is indicated by a straight line, whose slope gives the displayed value of the exponent *H* within error bars ±0.009.

The constraints of cognitive recognition at the level of tags affect the social process between actors as well as the structure of the co-evolving network. The time-series analysis is used to uncover prominent features of the coherent fluctuations in this process. We determine the fractal characteristics (see Methods) of the activity time series. In particular, we consider the time series of the number of all answers to the existing questions per time step as well as the time series of such events that contain a particular cognitive element. The results, Fig. 4, reveal that the clustering of events (avalanches) occurs as a distinguishing feature of self-organized processes. In addition, a high persistence is observed in the temporal fluctuations, both in the empirical data and simulations for a varied range of the agent’s expertise, Fig. 5. Measured by Hurst exponent (*H* > 0.5), a similar persistence was found in the processes of thematic discussions^15^. While somewhat lower Hurst exponents characterize the fluctuations in prototypal online social interactions^17^ and market dynamics^33^.

Several sequences of clustered events, determined (see Methods) from the corresponding time series, are reported in Fig. 4a. Considering a particular sequence, the avalanche size differences (returns) *d*_*λ*_ = *s*_*λ*+1_ − *s*_*λ*_, *λ* = 1, 2 · · · *λ*_*max*_ are found to exhibit non-Gaussian fluctuations. Fig. 4b shows the universal plot of the distributions for the appropriately scaled returns. It turns that the *q*-Gaussian expression *f*(*x*) = *a*[1 − (1 − *q*)(*x*/*b*)^2^]^1/(1−*q*)^, which was observed in a variety of complex dynamical systems^34^,^35^,^36^,^37^, well approximates these distributions (see Fig. 4 in SI). Interestingly, the values^36^ for the nonextensivity parameter *q* obtained in these cognitive-driven processes are higher compared with the corresponding parameter in emotion-driven social dynamics^15^.

The considered time series and the results of their fractal analysis for the empirical data and simulations are reported in Fig. 5a–d. Note that the rate of new arrivals in the empirical data, *p*(*t*), is also used as a creation rate of new agents in the simulation (see Methods). It exhibits distinct temporal correlations, which are carried out from the user’s real life. In this case, *p*(*t*) also shows an increasing trend that eventually yields the increase in the entire activity over time both in the empirical and simulated data, Fig. 5a,c. Hence, the detrended fractal analysis is performed, as described in Methods. Shown in Fig. 5b, the fluctuations in the number of answers containing all tags in the empirical data are characterized by the scaling exponent *H* = 0.85 ± 0.07. Similarly, persistent fluctuations with the exponents in the range *H* ∈ [0.62, 0.68] are found in the series of selected events that contain a particular tag. The results of an analogous analysis of the simulated data are shown in Fig. 5d. The time series of the number of answers with all tags and series containing a particular tag have the scaling exponents that are slightly higher, implying a stronger persistence, compared with the corresponding series of the empirical data. Here, we also consider temporal activity of three identified combinations of tags, three bottom curves, which exhibit a similar scaling behavior. These results show that the enhanced self-organization among actors emerges in the interactions with tag recognition, which is mandatory in the model, and, to a large extent, applies to the empirical data.

### Knowledge-sharing communities

**Figure 6:**
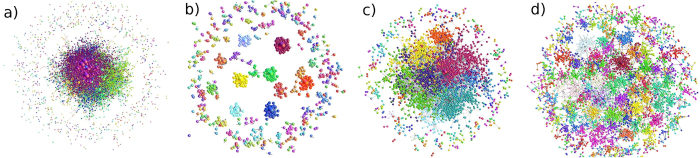
Community structure in bipartite networks of actors and questions reflecting the population of experts. Compressed bipartite network of actors and questions from Mathematics dataset (**a**) and simulation with the population of experts Exp1 (**b**) Exp2 (**c**) and in the case of ExpS but with non matching expertise *μ*-process (**d**). The observed communities, indicated by different colors, contain actors interconnected by questions. Each question node contains all answers related to it.

The coevolving bipartite networks, Fig. 6, emerge in various scenarios in the simulations and empirical data. Note that these networks are different from the single-question graph in Fig. 1b. In this case, each actor is a separate node while a compressed information on a particular question including all answers related to that question represents a single node of the question-partition. The structure of communities detected in these networks clearly stresses the importance of the actor’s expertise. In particular, in the case Exp1, the communities containing a specified single expertise grow as independent clusters, Fig. 6b. The situations when the agents have more than one expertise permit formation of larger communities of agents and questions. For a broad range of the agents’ expertise, the compact communities grow resembling the ones in the empirical data (see also Fig. 5 in SI). It is interesting to note that a dominant node representing a very active knowledgeable actor appears in each community. On the contrary, the pattern of communities is entirely different when the cognitive recognition does not drive the linking, Fig. 6d.

## Conclusions and outlook

Knowledge building via social interactions is studied as a collective phenomenon in an extended space—network of actors and their artifacts, where cognitive recognition interactions are active. We have considered an abundant empirical dataset with cognitive elements as mathematical tags and a two-scale dynamics modeling close to the data, which enabled a quantitative analysis of the process from the microscopic to global scale. Our approach permits to reveal the importance of each cognitive element, as well as the expertise of each actor and its activity pattern in the creation of the collective knowledge. Specifically, when the interacting actors possess a diversity of expertise, the process based on the meaningful (cognitive recognition) interactions leads to the innovation and the advance of knowledge of the emerging communities. When a broad spectrum of expertise is present in the population of actors, i.e. as in the empirical system, the process is quite efficient in creating the enlarged space where innovation can occur. In this case, the formation of coherent communities that share the knowledge is associated with the presence of several actors possessing a broad range of expertise. Notably fewer developed communities and a slower advance of knowledge characterize the population with a narrow distribution of expertise; entirely isolated communities and vanishing of innovation is found in the limiting case of a single expertise per individual. In contrast to the meaningful interactions, the case with ad hoc social linking leads to an entirely different outcome, even though, the individual actors possess a broad distribution of expertise. The advance of innovation measured at the system level appears fragmented in a variety of the emerging communities, each of which shares a limited amount of randomly accumulated knowledge.

The dynamics of social and cognitive elements, interwoven at the elementary scale, induces a type of self-organized process where several quantitative characteristics appear to be different from a prototypal social dynamics. Besides theoretical implications of our results in the study of cognitive-driven processes on networks, the presented approach can be directly applied in the analysis of other empirical systems that entail social collaborative efforts^19^,^20^. Examples include, but not limited to, social computing, crowdsourcing scientific knowledge production or scientific discovery games, and other emerging areas of increasing importance in the modern science and society^38^,^39^,^40^. The presented theoretical concept can prove to be useful in modeling physical systems at nanoscale^41^, for instance, the assembly of smart nanostructured materials with biological recognition.

## Methods

### Data structure

As a platform for scientific collaboration^23^, *Mathematics* is a part of *StackExchange: expert answers to your questions* network. For this work, the dataset was downloaded on May 5, 2014 from https://archive.org/details/stackexchange. It contains all user-contributed contents on *Mathematics* since the establishment of the site, July 2010, until the end of April 2014. Specifically, the considered dataset contains 77895 users, 269819 questions, 400511 answers and 1265445 comments. A detailed information is given about user id, the user’s activity (posting, answering, commenting), time stamp, list of tags for questions, and id of the corresponding question or answer to which a given answer or comment refers. The set of tags in answer/comment is inherited from the related question.

### Network mapping and topology analysis

Actions of users in Q&A dynamics are mapped onto a directed bipartite graph, where users, as one partition, interact indirectly via artifacts (questions, answers or comments), as another partition. At a user node *i* an incoming link is inserted to indicate that that user reads the corresponding artifact while an outgoing link stands for the user’s posting of a new artifact. The path of directed links from a question to a user to answer accurately describes the relationship of the answer to the original question, as it is included in the empirical data and strictly observed in the model. We also introduce a compressed bipartite network, where each question-node includes a question with all answers and comments related to that question; typically, they contain a larger number of tags thus expanding the original question’s attributes. The graphs layouts are done using **Gephi**; the community structure is detected by the maximum modularity algorithm^42^.

### The user’s activity and estimation of expertise

Assuming that a particular expertise of a user *i* is necessary to answer a given question (which is marked by a set of tags), we consider the amount of the user’s actions related to a particular tag, *κ*. Each tag that appears in the data is considered, in total 1040 tags. Hence, we compute a fraction 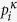 of the user’s actions *N*_*i*_ that is spent at *κ*-tag. For those tags where 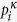 exceeds the average probability for that user, we set unity, indicating that the user *i* is an expert in these categories; thus, the user’s *i* expertise list is formed containing in total 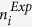 tags which received unity mark. The rest of tags receive zeros for that user. The entropy measure for each user, 
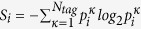
, remarkably quantifies the heterogeneity of the user’s expertise, both in answering and posting questions, Fig. 1e and Fig. 3b in SI, respectively. In the model, the agent’s expertise is specified from the list of 32 tags. Different populations of experts correspond to the situations where each agent gets a fixed number 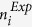 tags. In particular, one-tag expertise (Exp1), two-tags expertise (Exp2), four-tags expertise (Exp4), etc., correspond to the agent’s expertise list with two, four, etc. randomly selected tags. The case marked as ExpS is close to the empirical data, i.e., each agent gets a list of 2^*S*^ ≤ 32 tags, where the random number *S* is taken from the empirical distribution in Fig. 1e.

### Tag-related entropy

Following^28^, we define *T*_*j*_ as the time interval between the first occurrence of a tag *j* and the last activity in the dataset. Counting the total number of times *m* that the tag *j* occurred, we divide *T*_*j*_ into *m* equal subintervals. Then for each *i* = 1, · · · *m* we count the number of events *f*_*ji*_(*m*) related to the tag *j* in the *i*-th subinterval and compute the entropy *S*_*j*_(*m*) of the tag’s *j* sequence as 
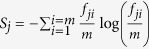
. For each tag in the dataset, the tag’s entropy normalized with the corresponding factor *log*(*m*) is represented by a point in Fig. 1f.

### Conditional probabilities of tag-related events

Four conditional probabilities appearing in Eqs (1) and (2), are defined and computed as follows: *P*(*κ*|*Q*^*K*^), probability that the *κ*-tag is present given the presence of questions *Q*^*K*^, is computed as the frequency of *κ*-tag in all questions; *P*(*A*^*K*^|*κ*, *Q*^*K*^), the probability that answers *A*^*K*^ exist given the questions *Q*^*K*^ with *κ*-tag, is given by the fraction of users whose expertise includes *κ*-tag of all active users in *K*th window; *P*(*A*^*K*^|*Q*^*K*^), the probability that answers *A*^*K*^ exist given the question *Q*^*K*^ (independently on the presence of *κ*-tag) is obtained as the ratio of the number of matching tags of all active users in *K*th window with all tags in the present questions; *P*(*κ*|*A*^*K*^, *Q*^*K*^), the probability to find the tag *κ* given the questions and answers in the *K*th window is determined from the above probabilities via chain relation.

### Definition of temporally clustered events

A cluster (or avalanche) represents a set of events enclosed between two consecutive drops of the time series to the baseline (noise level)^43^,^44^,^45^.

### Detrended time series analysis

To remove the local trend (an increasing activity and a weak 4-month cycle) appearing in the time series in Fig. 5, we apply the method of overlapping intervals^17^,^46^. Then, for each time series *h*(*k*), *k* = 1, 2, · · · *T*_*h*_ the profile 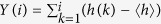 is divided into *N*_*s*_ segments of length *n* and the standards deviation around the local trend *y*_*μ*_(*i*) is computed at each segment *μ* = 1, 2 · · · *N*_*s*_, i.e., 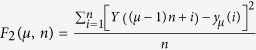. Varying the segment length *n*, the scale invariance 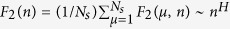 is examined to determine the Hurst exponent *H*.

### Model rules of interacting agents with expertise

Assuming that the new arrivals in the system boost the activity^47^, the agents are introduced with a pace *p*(*t*) agents per time step, where *p*(*t*) is the empirical time series of new users, shown in Fig. 5a. Each new agent receives a unique id and a fixed profile. The agents’ profiles statistically match the profiles of users in the data. Specifically, the agent’s activity level is set by the number of actions *N*_*i*_ ∈ *P*(*N*_*i*_), where *P*(*N*_*i*_) is the distribution of the user’s activity averaged over all users in the data (see SI:Fig. 3a). Subsequently, the agent’s probability *g*_*i*_ to post a question, or otherwise answer other questions, 1 − *g*_*i*_, is selected according to the interdependence *g*_*i*_ and *N*_*i*_ shown in Fig. 1c. Furthermore, the agent’s expertise is fixed by first setting the number of tags 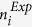, according to the considered situation, i.e. Exp1, Exp2, Exp4, or ExpS, and then making the list of the agent’s expertise of 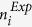 tags by random selection from the common list of 32 tags. The interactivity time of a new agent is set to Δ*T* = 0, which implies its immediate action. After each completed action, a new delay Δ*T* ∈ *P*(Δ*T*) is taken, where *P*(Δ*T*) is the empirical distribution for users, Fig. 1d. Note that both *p*(*t*) and *P*(Δ*T*) have the same temporal resolution, one bin representing 10 minutes in the original data. All agents are systematically updated, and the agents with an expiring delay time are placed in the *active agents* list. Each active agent, with its probability *g*_*i*_, puts a new question. Otherwise, it attempts to answer a question from the updated list of interesting questions. The list is created by considering all questions of next-neighbor agents on which an activity occurred within previous *T*_0_ = 10 steps. With a given probability that item can be searched elsewhere. In both cases, the agent’s action is the subject of the expertise matching. In the case of *μ*-process, with the probability *μ* = 0.5 an agent connects to a random question and post an answer while the matching of tags with the agent’s expertise is not required, but it can occur by chance (see illustration Fig. 1 in SI, and Algorithm in SI).

## Additional Information

**How to cite this article**: Dankulov, M. M. *et al.* The dynamics of meaningful social interactions and the emergence of collective knowledge. *Sci. Rep.* **5**, 12197; doi: 10.1038/srep12197 (2015).

## Supplementary Material

Supplementary Information

## References

[b1] Van Vliet C. M. Equilibrium and non-equilibrium statistical mechanics, 2nd edition, (World Scientific, New Jersey, 2010).

[b2] Itzykson C. & Drouffe J.-M. Statistical field theory Volume 1 & 2 (Cambridge University Press, Cambridge, 1989).

[b3] Balian R. From microphysics to macrophysics Volume I & II (Springer-Verlag, Berlin, 1991 & 1992).

[b4] Goldenfeld N. & Woese C. Biology’s next revolution. Nature, 445, 369 (2007). 17251963 10.1038/445369a

[b5] Goldenfeld N. & Woese C. Life is physics: evolution as a collective phenomenon far from equilibrium. Annu. Rev. Condens. Matter Phys. 2, 375–399 (2011).

[b6] Tunstrom K. *et al.* Collective states, multistability and transitional behavior in schooling fish. PLoS Comput. Biol. 9, e1002915 (2013). 23468605 10.1371/journal.pcbi.1002915 PMC3585391

[b7] Cavagna A. & Giardina I. Birds flocks as condensed matter. Annu. Rev. Condens. Matter Phys. 5, Ed. Langer J. S. , 183–207 (2014).

[b8] Attanasi A. *et al.* Information transfer and behavioural inertia in starling flocks. Nature Phys. 10, 691–696 (2014). 10.1038/nphys3035 PMC4173114 25264452

[b9] Orlandi G. J., Soriano J., Alvarez-Lacalle E., Teller S. & Casademunt J. Noise focusing and the emergence of coherent activity in neuronal cultures. Nature Phys. 9, 582–590 (2013).

[b10] Conte R. *et al.*, Manifesto of computational social science, Eur. Phys. J. Special Topics 214, 325–346 (2012).

[b11] Carpendale J. I. M. & Müller U. Editors, Social Interactions and the Development of Knowledge (Lawrence Erlbaum Associates, Inc. Mahwah, New Jersey, 2013).

[b12] Kenrick D. T., Li N. P. & Butner J. Dynamical evolutionary psychology: Individual decision rules and emergent social norms. Psychol. Rev. 110, 3–28 (2003). 12529056 10.1037/0033-295x.110.1.3

[b13] Loretto V. & Steels L. Emergence of language. Nature Phys. 3, 758–760 (2007).

[b14] Thurner S., Szell M. & Sinatra R. Emergence of good conduct, scaling and Zipf laws in human behavioral sequences in an online world. PLoS ONE 7, e29796 (2012). 22253784 10.1371/journal.pone.0029796 PMC3257232

[b15] Tadić B., Gligorijević V., Mitrović M. & Šuvakov M. Co-evolutionary mechanisms of emotional bursts in online social dynamics and networks. Entropy 15, 5084–5120 (2013).

[b16] González-Bailón S., Borge-Holthoefer J., Rivero A. & Moreno Y. The dynamics of protest recruitment through an online network. Sci. Rep. 1, 197 (2011). 22355712 10.1038/srep00197 PMC3240992

[b17] Šuvakov M., Mitrović M., Gligorijević V. & Tadić B. How the online social networks are used: dialogues-based structure of myspace. J. R. Soc. Interface 10, 20120819 (2012). 23193108 10.1098/rsif.2012.0819 PMC3565699

[b18] von Scheve Ch. & Salmela M. Editors Collective emotions (Oxford University Press, 2014).

[b19] Boudreau K., Gaule P., Lakhani K. R., Riedl Ch. & Woolley A. From crowd to collaborators: initiating effort and catalyzing interactions among online creative workers. Harvard Business School, Working paper 14-060, (2014), http://nrs.harvard.edu/urn-3:HUL.InstRepos:12111352.

[b20] Lakhani K. R. & von Hippel E. How open source software works: “free” user-to-user assistance. Res. Policy, 32, 923–943 (2003).

[b21] Kimmerle J., Kress U. & Held Ch. The interplay between individual and collective knowledge: technologies for organisational learning. Knowl. Manag. Res. Pract. 8, 33–44 (2010).

[b22] Kitchener R. F. Piaget’s social epistemology. Social interactions and the development of knowledge, Carpendale J. I. M. & Müller U. Editors (Lawrence Erlbaum Associates, Inc.: Mahwah, New Jersey, , pp. 45–66, 2013).

[b23] Nielsen M. Reinventing discovery: The new era of networked science (Princeton University Press, 2012).

[b24] Bowker G. C., Leigh Star S., Turner W. & Gasser L. Editors, Social science, technical systems, and cooperative work, (Psychology Press, New York, 2014).

[b25] Baez J. C. Math Blogs. Notices Amer. Math. Soc. 333 (2010).

[b26] Youn H., Bettencourt L. M. A., Strumsky D. & Lobo J. Invention as a combinatorial problem: evidence from US patents. *arxiv:1406.2938* (2014). 10.1098/rsif.2015.0272 PMC4424706 25904530

[b27] Thurner S., Klimek P. & Hanel R. Schumpeterian economic dynamics as a quantifiable minimum model of evolution. New J. Phys. 12, 075029 (2010).

[b28] Tria F., Loreto V., Servedio V. D. P. & Strogatz S. H. The dynamics of correlated novelties. Sci. Rep., 4, 5890 (2014). 25080941 10.1038/srep05890 PMC5376195

[b29] Font-Clos F., Boleda G. & Corral Á. A scaling law beyond Zipf’s law and its relation to Heaps’ law. New J. Phys. 15, 093033 (2013).

[b30] Grendar M. & Niven R. K. The Pólya information divergence. Inform. Science 180, 4189–4194 (2010).

[b31] Niven R. K. Combinatorial entropies and statistics. Eur. Phys. J. B 70, 49–63 (2009).

[b32] Hsieh P.-H. A nonparametric assessment of model adequacy based on Kullback-Leibler divergence. Stat. Comput. 23, 149–162 (2013).

[b33] Alvarez-Ramirez J., Alvarez J., Rodriguez E. & Fernandez-Anaya G. Time-varying Hurst exponent for US stock market. Physica A 387, 615–6169 (2008).

[b34] Tsallis C. The nonadditive entropy Sq and its applications in physics and elsewhere: Some remarks. Entropy 13, 1765–1804 (2011).

[b35] Tsallis C. & Gell-Mann M. Editors. Nonextensive entropy—interdisciplinary applications (Oxford University Press, Oxford, 2004).

[b36] Pavlos G. P. *et al.* Universality of Tsallis non-extensive statistics and fractal dynamics of complex systems. Chaotic Mod. Simul. (CMSIM) 2, 395–447 (2012).

[b37] Caruso F., Pluchino A., Latora V., Vinciguerra S. & Rapisarda A. Analysis of self-organized criticality in the Olami-Feder-Christensen model and in real earthquakes. Phys. Rev. E 75, 055101(R) (2007). 10.1103/PhysRevE.75.055101 17677120

[b38] Fortino G., Galzarano S., Gravina R. & Li W. A framework for collaborative computing and multi-sensor data fusion in body sensor networks, Inform. Fusion 22, 50–70 (2015).

[b39] http://www.gameslearningsociety.org/ Games Learning Society, (2013) Date of access: 14/05/15.

[b40] http://www.complex-systems.meduniwien.ac.at/events/insite13/ INSITE Workshop: Games, Science & Society, (2013) Date of access: 14/05/15.

[b41] Badu S. R. *et al.* Modeling of RNA nanotubes using molecular dynamics simulations. Eur. Biophys. J. 43, 555–564 (2014). 25208764 10.1007/s00249-014-0985-6 PMC6345520

[b42] Lancichinetti A., Kivela M., Saramäki J. & Fortunato S. Characterizing the community structure of complex networks. PLoS ONE 5, e11976 (2010). 20711338 10.1371/journal.pone.0011976 PMC2920821

[b43] Tadić B. Nonuniversal scaling behavior of Barkhausen noise. Phys. Rev. Lett. 77, 3843–3846 (1996). 10062322 10.1103/PhysRevLett.77.3843

[b44] Spasojević D., Bukvić S., Milošević S. & Stanley H. E. Barkhausen noise: elementary signals, power laws, and scaling relations. Phys. Rev. E 54, 2531 (1996). 10.1103/physreve.54.2531 9965364

[b45] Mitrović M., Paltoglou G. & Tadić B. Quantitative analysis of bloggers’ collective behavior powered by emotions. J. Stat. Mech. Theor. Exp. 2011(02), P02005 (2011).

[b46] Hu J., Gao J. & Wang X. Multifractal analysis of sunspot time series: the effects of the 11-year cycle and Fourier truncation. J. Stat. Mech. Theor. Exp. 2009(02), P02066 (2009).

[b47] Mitrović M. & Tadić B. Dynamics of bloggers’ communities: Bipartite networks from empirical data and agent-based modeling Physica A. 391, 5264–5278 (2012).

