# Supplementary material for: The dynamics of meaningful social interactions and the emergence of collective knowledge
Source: Sci Rep. 2015 Jul 15;5:12197. doi: 10.1038/srep12197 (PMC4502430; doi:10.1038/srep12197)
Supplement: Supplementary Information [file srep12197-s1.pdf]

Supplementary information: The dynamics of meaningful social interactions and the emergence of collective knowledge

Marija Mitrović Dankulov[a,b], Roderick Melnik[c] and Bosiljka Tadić[b]

[a] *Scientific Computing Laboratory; Institute of Physics Belgrade; University of Belgrade; Belgrade; Serbia*

[b] *Department for Theoretical Physics; Jožef Stefan Institute; Ljubljana; Slovenia*

[c] *MS2Discovery Interdisciplinary Research Institute; M2NeT Laboratory and Department of Mathematics; Wilfrid Laurier University; Waterloo; ON; Canada*

Appearance of tags combinations depends on the actors' expertise, and their activity, Fig.SI1.

Ranking distribution of tag frequencies, both at individual tags level as well as at the level of tags combinations, exhibits a broad range (Zipf's laws) compatible with the collective dynamics of tags and innovation (Heaps' law), Fig.SI2.

Users' heterogeneity is quantified by the power-law distribution of the activity level N_i, Fig.SI3a. In addition, the same user has a different level of activity when posting and answering questions. The entropies computed from the posting-question events results in a different distribution, Fig.SI3b, compared with the distribution of the answering-question events, which is shown in Fig. 1e in the paper.

q-Gaussian distribution fits for the avalanche returns in different scenarios, described in the text, Fig.SI4.

Communities of actors and their posts–questions and answers are observed both in the empirical data and in simulations, Fig.SI5:

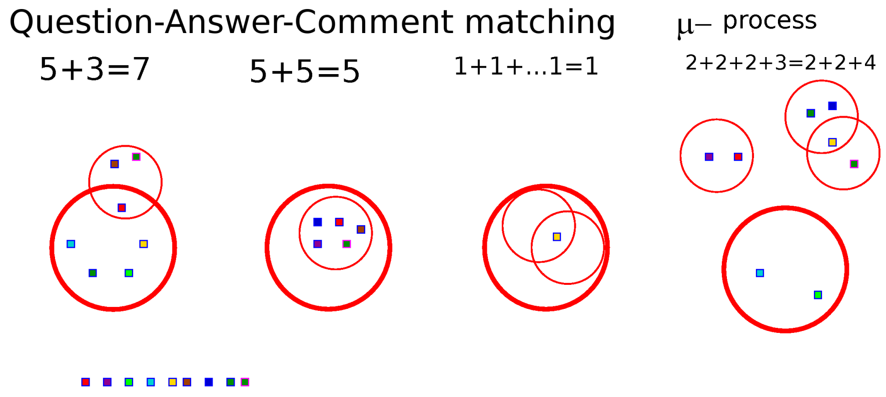

Figure 1: **Illustration of the combinatorial processes of tags.** Appearance of new combinations of tags in the process where tag-matching between a question and its answers is required (left part), and spontaneous matching in μ-process (right). In each "reaction" equation, the right-hand side indicates the number of different tags that are connected to the emerging combination. Squares of different colors represent different tags that are present in the process.

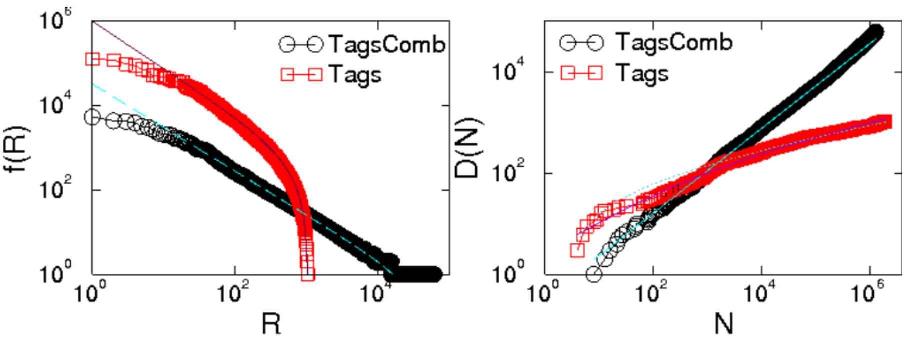

Figure 2: **Zipf's and Heaps' law for the appearance of tags and their combinations in the empirical dataset.** Frequencies of individual tags are compared with tags-combination frequencies. Note that the total number of the identified tags is 1040 while the number of potential combinations is virtually unlimited.

(a) (b)

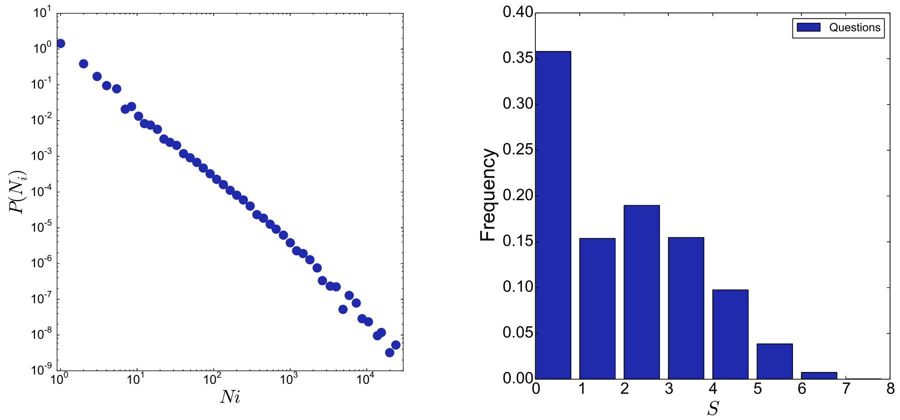

Figure 3: **In the empirical data, users differ from each other in their activity patterns and knowledge.** The distribution of the number of events per user (a), and the distribution of entropy from the posting-question events per user (b), averaged over all users in the empirical data.

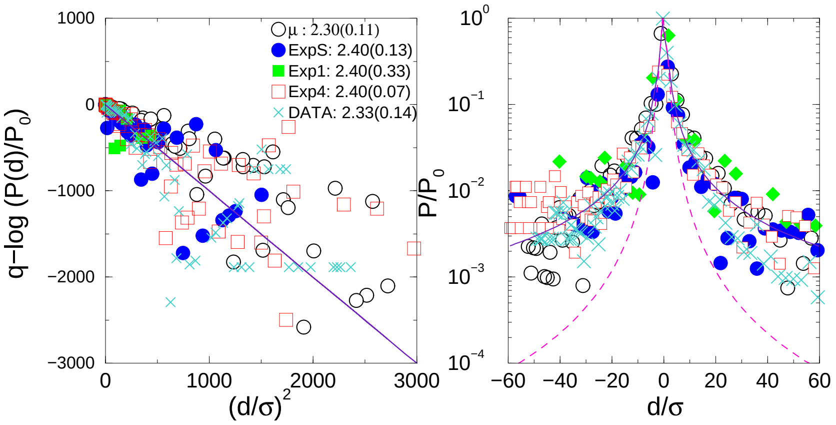

Figure 4: **Returns' q-log plot.** Testing the linear dependence of q-logarithm of the distributions against $(d/\sigma)^2$ for the indicated q-value within the quoted error bars.

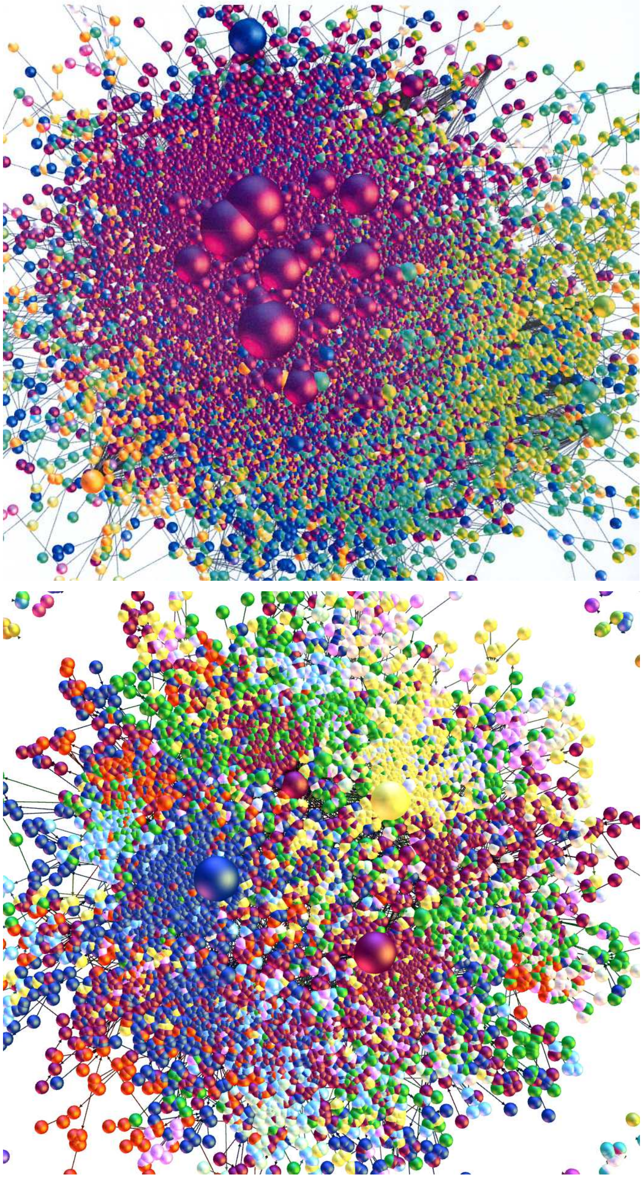

Figure 5: **Communities in a bipartite network are formed around big actors.** (top) Network from empirical data of a one-month activity; (bottom) Growing communities in the simulations, the case with ExpS distribution of expertise. Detected communities are marked by different colors.

Algorithm 1 Program flow for agent-based model of cognitive interactions

INPUT: Time series $p(t)$; Distributions $P(\Delta T)$, $P(N_i)$, $g_i(N_i)$, $P(n_i^{Exp})$; N_t;
Start empty lists LAU (active agents), LAQ (active questions) and LIQ (interesting questions);
for all $t = 1, \dots, N_t$ **do**
  for all $i = 1, \dots, p(t)$ **do**
    Add new agent; Assign $N_i \in P(N_i)$, $g_i : N_i$, n_i^{Exp}, L_i^{Exp}, $m_i = 0$, $\Delta T = 0$;
    Add i to the list of active agents (LAU);
  end for
  for all $i \in LAU$ **do**
    if g_i **then**
      Agent i asks a new question k; Question k is assigned to $1 \leq c \leq 5$ tags, randomly selected from the categories in which the agent i has expertise;
      Update bipartite network;
    else
      if $!\mu$ **then**
        Form the list LIQ of potentially interesting questions for the agent i: among all questions linked to the agent's i next-neighbor agents select those on which an activity occurred within the last T_0 steps;
        Select a question $j \in LIQ$; Agent i posts an answer to question j if the condition of expertise matching is satisfied; clear LIQ;
        Update bipartite network;
        With probability 0.5 agent i selects a question from LAQ and posts an answer if it matches the agent's i expertise;
        Update bipartite network;
      else
        Agent i selects and answers a question from LAQ (regardless the agent's i expertise);
        Update bipartite network;
      end if
    end if
    Update the agent's action counter m_i;
    Select the agent's new delay time $\Delta T \in P(\Delta T)$;
  end for
  For all other agents: Reduce the agents' delay time $\Delta T - -$;
  Update LAQ by all questions on which an activity occurred in the previous T_0 time steps;
  Along the connections to the active questions list prompt the agents that have positive delay time by selecting a new $\Delta T \in P(\Delta T)$;
  Remove each agent i whose number of actions m_i reached its maximum N_i.
  Update LAU with agents whose current delay time is zero;
end for
END
